# Public Health Responses to a Dengue Outbreak in a Fragile State: A Case Study of Nepal

**DOI:** 10.1155/2013/158462

**Published:** 2013-04-03

**Authors:** Karolina Griffiths, Megha Raj Banjara, T. O'Dempsey, B. Munslow, Axel Kroeger

**Affiliations:** ^1^Liverpool School of Tropical Medicine, Pembroke Place, Liverpool L3 5QA, UK; ^2^Central Department of Microbiology, Tribhuvan University, Kathmandu, Nepal; ^3^Special Programme for Research and Training in Tropical Diseases (TDR), World Health Organization, 20 Avenue Appia, 1211 Geneva 27, Switzerland

## Abstract

*Objectives*. The number of countries reporting dengue cases is increasing worldwide. Nepal saw its first dengue outbreak in 2010, with 96% of cases reported in three districts. There are numerous policy challenges to providing an effective public health response system in a fragile state. This paper evaluates the dengue case notification, surveillance, laboratory facilities, intersectoral collaboration, and how government and community services responded to the outbreak. *Methods*. Qualitative data were collected through 20 in-depth interviews, with key stakeholders, and two focus-group discussions, with seven participants. *Results*. Limitations of case recognition included weak diagnostic facilities and private hospitals not incorporated into the case reporting system. Research on vectors was weak, with no virological surveillance. Limitations of outbreak response included poor coordination and an inadequate budget. There was good community mobilization and emergency response but no routine vector control. *Conclusions*. A weak state has limited response capabilities. Disease surveillance and response plans need to be country-specific and consider state response capacity and the level of endemicity. Two feasible solutions for Nepal are (1) go upwards to regional collaboration for disease and vector surveillance, laboratory assistance, and staff training; (2) go downwards to expand upon community mobilisation, ensuring that vector control is anticipatory to outbreaks.

## 1. Introduction

Dengue is the most important vector-borne viral infection worldwide with 2.5 billion people at risk, according to the World Health Organisation (WHO) [1]. The number of cases and number of countries affected has doubled from the period 1990–1994 to 2000–2004 [1–3]. Larger epidemics are becoming more common [4], with countries in Asia witnessing an increase in severe dengue and a higher mortality in the earlier stages [5].

Nepal reported its first case of dengue in 2004 and the first indigenous case in 2006 [6]. Only sporadic cases were then seen until the outbreak in 2010, with 917 cases reported nationally (unpublished data), seen almost solely in 3 administrative districts in the Terai region on the Indian border: Chitwan, Nawalparasi, and Rupandehi. The aim of this paper is to describe the public health surveillance and outbreak response to dengue in Nepal in 2010 and to draw lessons from the experience of a fragile state.

The WHO World Health Assembly urged member states to improve surveillance, prevention, control, and management of dengue [7]. Guidelines highlight the need to incorporate lessons learnt from country experiences into outbreak response plans [1]. Country responses to dengue have been documented from all around the world [8]; however, little published literature is available in countries which have seen a recent introduction of dengue viruses, such as Nepal. This research seeks to address the gap.

 The “Early Warning Reporting System” (EWARS) in Nepal was set up in 1997 with eight hospital sentinel sites rising to 39 hospital sentinel sites by 2011. There are six targeted diseases/syndromes: malaria, kala-azar, dengue fever, acute gastroenteritis, cholera, and severe acute respiratory infection. Nepal is a postconflict fragile state, a term used “*for countries facing particularly severe development challenges: weak institutional capacity, poor governance and political instability*” [9]. Nepal faces many challenges in healthcare provision including weak, poorly structured healthcare services with financial and human resource limitations [10, 11]. Looking through the lens of dengue helps identify the key strengths and weaknesses in the public health system in the context of a fragile state. This paper focuses on dengue case notification, surveillance, laboratory facilities, intersectoral collaboration, and how government services and the community responded to the dengue outbreak.

## 2. Methodology

The study aims to describe the good practices and limiting factors of the surveillance “*recognition*” system and the intersectoral “*response*” to dengue.

### 2.1. Data Collection Tools and Sampling

Qualitative data collection tools were used to analyse the dengue outbreak response. Participants were sampled purposively, with deliberate selection regarding their experience, to help ensure involvement of the key stakeholders in the dengue surveillance and outbreak response, including government policy-makers, officials from three key districts, healthcare workers and community leaders. This range of perspectives ensures fair dealing, improving the quality of the data. A list of all government and district officials and healthcare providers involved with the dengue surveillance and outbreak response was compiled with help from Epidemiology and Disease Control Division (EDCD) and Public Health and Infectious Disease Research Centre in Nepal—this became the sampling frame. Further participants were added to this by snowball sampling.  Table 1 identifies the number and roles of the participants. All central officials involved with dengue were included plus district officials from the 3 districts most affected, Chitwan, Nawalparasi, and Rupandehi. Doctors from the central level and all three districts that had managed dengue cases were included. Other districts with few cases were excluded (<4% of total dengue cases). Community leaders available for focus groups discussions (FGDs) were identified with district officials. Contact was made with each person involved via phone or in person. The sample size took into account the saturation principle; data collection was deemed sufficient when new data did not provide new information or ideas on the topics being questioned.

### 2.2. Data Collection

In-depth interviews and focus group discussions were used for data collection. These were conducted four to five months after the outbreak had subsided. 

#### 2.2.1. In-Depth Interviews

Twenty in-depth interviews explored the experiences, successes, and failures of the recognition of dengue cases and response to the outbreak. The brief topic guide used is shown below. This was elaborated on, allowing flexible questioning and exploration of issues raised.

- Recognition of the Dengue Cases
  - Which disease surveillance techniques are used and how data surveillance is analysed?
  - How is dengue classified?
  - How are dengue cases reported?
  - The laboratory confirmation available.
  - Collecting data for virological surveillance and its use.
  - Vector surveillance—what surveys are done?
- Response to the Dengue Outbreak
  - Vector control—how is this done and by whom?
  - Community participation—to what extent is the community involved and how?
  - Preparing for a dengue outbreak—is there a plan?
  - Recent experience with a dengue outbreak.
  - Which sectors and organisations were involved with outbreak response, including training and measures used?
  - Budget.

#### 2.2.2. Focus Group Discussion

A larger FGD was run with five doctors in a district hospital. A small focus group was held with community leaders.

### 2.3. Data Analysis

Qualitative data collected through in-depth interviews and FGDs were transcribed, coded, and analysed using NVIVO software.

### 2.4. Ethical Aspects

Each participant provided informed consent for data collection and recordings. The research received ethical approval from the Liverpool School of Tropical Medicine and the Nepal Health Research Council (Reference no. 981, 2011).

## 3. Results

Tables 2 and 3 summarise the main activities and limitations regarding dengue outbreak detection and response described by participants, alongside potential recommendations suggested. 

### 3.1. Recognition of the Dengue Cases

Guidelines on the dengue surveillance system and dengue clinical management were available but there were no other public health guidelines. With one exception, all respondents recognised that dengue reporting was mandatory.

Sentinel site surveillance was identified as the principal surveillance method by 9 respondents. Ten respondents noted that active surveillance was undertaken; however it became clear from the interviews that this only occurred during an outbreak.

*“We have active data collection, only in case of outbreak”* (Government Official, Identifier 9). **

Eight out of 17 respondents noted that fever surveillance was undertaken. Nine participants noted that there was focal surveillance (active case detection around reported cases). However this only happened in two of the districts during the outbreak, not routinely. 

There were concerns that this surveillance system is inadequate. One respondent reported that “*we are only touching the tip of the iceberg*” as many fever cases will not present to hospital, and there may be “*hundreds of thousands of dengue cases*” in the community (Doctor in District Hospital, Identifier 16).

Furthermore, quality control of disease surveillance was insufficient.

*“We do not say if the data is accurate or not. We just analyse which hospitals, which sentinel sites send the data and how many cases are there”* (Government Official, Identifier 9). **

The late recognition of dengue cases by the government was noted to be the primary limitation. There is poor linkage of private sector hospitals into the national reporting system.

*“Most people go to [the] private sector… but they do not record or report properly. And less people go to visit the government clinics but there is a good system of recording.”*

**District Official, Identifier 22**

The main method of classifying dengue cases was the “DF/DHF/DSS” system (according to 14 participants who knew about classification). However, the majority of these participants (10 out of these 14) were aware of the revised WHO dengue case classification [1]. A national case definition of dengue is available, but clinicians did not consistently apply this, with a variety of answers provided when asked to state this definition.

Viral surveillance was not available in Nepal. A Government official said:

*“So many samples were collected here, [we] cannot identify the types of virus involved. We do not have the facility for virus isolation”* (Government Official, Identifier 24). **

#### 3.1.1. Laboratory Diagnostics

Interviews with healthcare workers and the laboratory technician highlighted that the main diagnostic tools were haematological and clinical findings alongside serological diagnosis (rapid diagnosis test (RDT) or MAC ELISA (IgM antibody capture enzyme-linked immunosorbent assay kit)). Only one participant had access to polymerase chain reaction (PCR) privately, which was not otherwise available. The lack of facilities within Nepal was identified as a key limitation by both government and clinical staff as ELISA tests were not available countrywide.

On analysis of secondary government data, at least 803 out of the 917 dengue cases nationwide were confirmed by IgG/IgM tests (either RDT or ELISA, not specified). There was a variation in the proportion of cases confirmed by the laboratory, with participants noting that between 10 and 50% of dengue cases were confirmed.

Regional healthcare centres relied on less specific and sensitive RDTs which were in short supply.

*“During the last season the government provided around 100 [RDT] kits in our hospital, and 100 kits will finish in one day. We see more than 200 people in one day!”*

**(Clinician, District Level, Identifier 20).**

#### 3.1.2. Budget

According to a senior health official, the overall budget for all dengue activities was $70'000 (US dollars) in 2010. These funds were used for the provision of RDT kits, orientation (one day training given to staff), and case management training. The central government provided all funds with no external assistance. Hence an “*insufficient budget*” at the district level was identified as the “*main problem*” (District official, Identifier 10).

### 3.2. Response

#### 3.2.1. Vector Surveillance before and during the Outbreak

Vector surveillance was poor, with no routine entomological surveys. Furthermore, these surveys were not conducted countrywide. One entomologist (Identifier 4) admitted that there were methodological weaknesses with “*no sampling method.*” The latest survey from 2010 was small and only examined 122 houses in one municipality. Respondents noted that this deficiency was due to “*a lack of human resources*” (Government Official, Identifier 6) and training. 

#### 3.2.2. Routine and Emergency Search and Destroy Campaigns

All vector control measures were done exclusively as an emergency response once the outbreak had started.

*“No experience among our health workers and local people [meant that] it took some time to realise this was not a disease but a disaster type of disease. It took two months to realise [that it was an outbreak of dengue]” (Government Official, Identifier 2).*

Key emergency vector control activities included community education and awareness campaigns, clean-up campaigns, environmental habitat destruction (“search and destroy” campaigns), and insecticide space spraying (fogging). 

**Figure 1:**
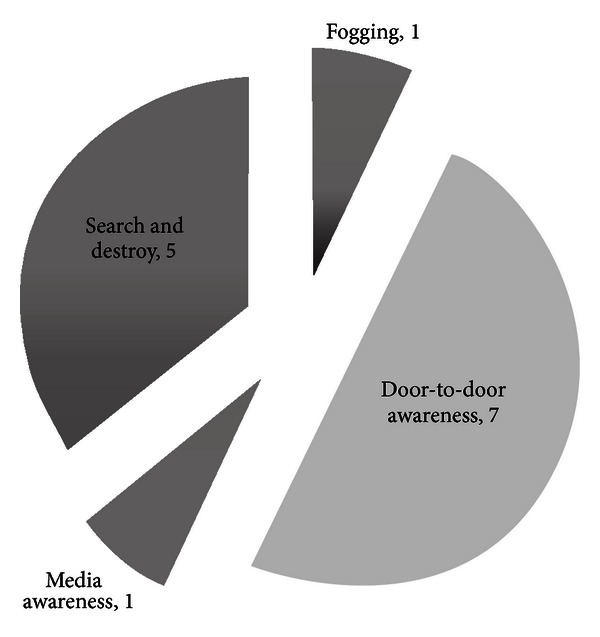
What was the most successful measure in the outbreak response? (Number of participants.) Responses of 14 Key respondents.

According to participants (Figure 1), the most successful response measures were the door-to-door awareness campaigns and the search and destroy campaign, which were run simultaneously by Female Community Health Volunteers (FCHVs). This included demonstrating the destruction of vector habitats and the use of water container covers. Furthermore, one respondent identified these FCHVs as the strongest partners in the outbreak response as “*community people believe them more than others*” (District Official, Identifier 19).

No respondent noted water treatment by insecticides or that insecticide-treated nets were in use. There was no process employed to improve water protection or cleaning. Despite recognising that fogging *“adversely affects our health” (District official, Identifier 10)*, it was still employed by district officials due to public demand. 

### 3.3. Intersectoral Cooperation and Coordination during the Outbreak

The emergency response was a good example of good intersectoral collaboration with many sectors being involved:

*“…hospitals as well as civil society, administrations, security forces, all sector people helped, even housewives, schoolchildren, market people, business people…” (Government Official, Identifier 9).*

However there was poor coordination between the central level and the periphery. There was confusion over which agency was in charge of vector control. Nine respondents thought that the district public health offices were in charge, six thought that the central level staff and one person thought the Vector borne disease research and training centre were in charge. The need for better coordination was recognised and plans to “*conduct an annual meeting of municipalities*” (District Official, Identifier 14) were in place. 

The media played a strong role in the outbreak; 16 respondents first heard about the outbreak through television or newspaper.

## 4. Discussion

The study findings demonstrate the extensive limitations existing in a fragile state that need to be addressed before and during a dengue outbreak in order to provide reliable dengue surveillance data and an adequate response in accordance with international dengue guidelines. Overall the response to dengue was lacking in numerous areas, such as disease surveillance, but was strong with respect to community participation and the media. The main issues encountered are discussed as follows.

### 4.1. Dengue and Disease Surveillance

#### 4.1.1. Case Notification

Good case notification relies on three key components: an accurate case definition, reliable diagnostic facilities, and quality control to assess the data. Case definition needs to be specific and applied consistently to prevent under-/overreporting of the epidemic and misdiagnosis. The majority of reported cases in Nepal were confirmed by IgG/IgM tests demonstrating that the outbreak data is valid. However, some respondents thought that up to 90% of potential dengue cases were not confirmed by a laboratory test. There may be a large number of potential cases undetected in the community, demonstrating the underrepresentation of true epidemic figures. 

Dengue guidelines state that as a minimum, laboratories should be able to perform IgM antibody-capture ELISA [1]. Relying on clinical and haematological features for a diagnosis can lead to a delay in laboratory diagnosis and response time. This weakness of diagnostics only available at central level has been documented in other countries such as Brazil [12]. Although, it is important to consider the context, improving access to reliable RDT kits across the country may be a practical alternative to providing more expensive facilities.

#### 4.1.2. Disease Surveillance

Various limitations of the surveillance systems can be highlighted. Active case detection, to complement passive surveillance, was only undertaken in two districts. Widespread active surveillance needs to be put in place to help predict future epidemics [13]. Surveillance should be continuous to collect information on the origin and distribution of dengue cases; helping to estimate incidence and allowing services to prepare. 

Sentinel surveillance can help determine transmission dynamics of infectious diseases; however as the sites only represent a specific cohort and not the general population, they cannot be used alone to ascertain national trends [1]. Thirty-nine sites in Nepal were apparently too few in such a diverse country with distinct terrains to detect the outbreak in a timely manner. 

Private hospitals not being properly integrated in the surveillance system were a key cause of notification delay, generally a problem in Nepal [11]. Many dengue patients in the high risk areas utilised private healthcare services—for example, 324 out of 592 dengue patients recorded in Chitwan/Nawalparasi were seen in just one private hospital. 

Studies from South East Asia have highlighted the underreported burden of dengue from hospital case reporting, with the average underrecognition of total dengue cases 8.7-fold in Thailand and 9.1-fold in Cambodia [14]. Hence, primary healthcare centres should be included in the surveillance system, as recommended previously in 2004 [15]. FCHVs are an effective part of the district healthcare system and should be better incorporated into the reporting system. 

Any changes in the surveillance system must recognise the cost and resource limitations. Including dengue surveillance as part of an integrated system for other vector-borne diseases may reduce costs and staffing. Although active surveillance increases the sensitivity of a surveillance system, it places huge demands on resources. Only Cuba and Singapore utilise a regular countrywide active surveillance programme [16]. There is little information on cost effectiveness of surveillance programmes and this needs to be addressed.

#### 4.1.3. Vector Surveillance

There is a huge research gap on vectors in Nepal, particularly with up-to-date dengue vector surveillance and control. Nepal cannot combat dengue without information on the vectors present, their spatial distribution, and environmental risk factors. Nepal has numerous other vector-borne and infectious diseases to contend with, for example, updating 40-year-old data on malaria prevalence. However, dengue should be a priority as new research could help prepare integrated response measures to mitigate the impact of future outbreaks.

Regional collaboration with neighbouring countries endemic for dengue, such as India, may facilitate this vector research through cost and expertise efficiencies, helping to implement a cross-border disease and virological surveillance programme and to provide comprehensive training. As another arboviral disease, a cross-border virological surveillance programme that includes Japanese encephalitis may prove beneficial for disease monitoring and research purposes.

### 4.2. Response to Dengue Outbreaks 

#### 4.2.1. Community Participation

The success of the awareness programmes and search and destroy campaigns can be attributed to excellent community mobilisation. Conflict may have had positive effects on health services in Nepal, notably helping to acknowledge community groups and civil society in health programmes [17]. This is in contrast to other literature which notes that mobilisation may not be suitable for communities in conflict [18]. However, evidence shows how conflict encouraged the development of community support programmes and FCHV involvement [19]. 

There are many reasons for building upon this good social mobilisation. Evidence suggests that community participation is vital in an outbreak response, both with dengue for vector control and reducing larval indices and with other vector-borne diseases [8, 20, 21]. However, a comprehensive review calls for better documentation of the successes of community participation, with weak evidence that the strategies have an effect on dengue transmission [20]. Future research needs to look at statistical evidence.

Both awareness and search and destroy programmes need to be routine, utilising community mobilisation and incorporating dengue in current job titles within the local district public health offices. Improvements in community participation should incorporate all areas vulnerable to dengue, not just selecting few districts. Sustainability of the programmes needs to be guaranteed and the community should be involved with decision-making. A more formal community coordination group needs to be created between different sectors such as water and sanitation. Such “*community working groups*” as seen in Cuba were in charge of all aspects, from designing plans, voicing community concerns, and evaluating results [22]. This highlights how communities can become self-sufficient—yet they will need some initial direction to build capacity [23]. This capacity building may come from the private sector or the government if the context allows.

#### 4.2.2. Improving Intersectoral Coordination

Consistent with dengue guidelines, a “Multisectoral Dengue Action Committee” should meet regularly, collaborating with sanitation services and government heads of environment to improve water supply [1]. Officials should liaise with nongovernmental organisations (NGOs) working in vector-borne diseases, such as malaria, to incorporate dengue into their programmes. 

#### 4.2.3. Role of the Media

The media played an important part in the outbreak response. Using the media for health communication has many benefits; it can be participatory and driven by the local context and culture allowing community members to be seen as equal [24]. For example, the reach of a mass media family planning campaign increased to 75% when indirect effects (spread of information through the community) were considered [25]. Mass media messages can have extensive impact in Nepal and should be built upon further.

### 4.3. Limitations of the Study

One limitation of the sampling methodology was not including more community leaders and members and FCHVs involved with the response. However, the sample included key stakeholders and policy makers in the government and the district. It was not possible to accurately determine the number of doctors involved in the dengue response; however the doctors from four hospitals that dealt with 81% of recorded cases were included (EDCD unpublished data 2010). 

 “Social desirability” of the participants was a limitation. The respondents may have been less willing to criticise themselves or their institutions in order to show themselves in the best light, according to social acceptability norms. Several times participants did not wish to comment about the Ministry of Health activities, reducing reliability of results. 

The role of the participant was not deemed to have induced any additional bias to results. The researcher was trained in qualitative research techniques to reduce introducing bias.

This research is not transferable to all other settings where dengue outbreaks are seen, such as wealthier countries or those with high dengue endemicity. However, this research provides a good insight into challenges dealing with emerging infectious diseases within a fragile political context.

## 5. Conclusion

It is difficult to run a disease surveillance system in a postconflict environment with reduced access to care and no consistent use of national case classification, particularly in a country like Nepal, where dengue has recently emerged. The political context can affect the financial and trained human resources available to combat dengue. Furthermore, low levels of endemicity require different strategies for dengue surveillance [16]. Future studies for the implementation of dengue surveillance and control programmes need to take into account the political context as well as the level of endemicity. 

**Figure 2:**
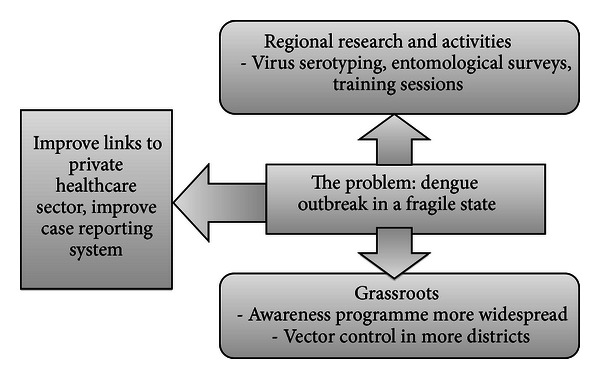
Realistic solutions to dengue: regional collaboration and community participation. Also need to improve case reporting system.

Country-specific packages for dengue outbreak response are needed which recognise realistic solutions that are achievable in the short term (Figure 2). Two key solutions involve the following:

1. using regional assistance to improve research into the vectors and spatial distribution of serotypes as well as to provide training for healthcare and public health professionals; regional efforts are advocated by many countries for improved vector control, including those with a strong political infrastructure, such as Singapore [26];
2. building upon good community mobilisation and improving awareness; potential strategies include FCHV help with primary care case notification, wider participation in vector control activities, and the quick mobilisation of community response teams.

Alongside this, the need for the integration of a case reporting system in the private sector must be addressed as a priority. With good intersectoral and regional collaboration, community empowerment, and access to reliable diagnostic facilities, major dengue outbreaks can be prevented.

## Figures and Tables

**Table 1:** Number and role of participants involved in each component of the research.

Component of study	Number of participants	Role of participants
In-depth interview evaluating dengue response	20	6 government officials, 2 entomologists, 9 district officials (from District Public Health Office, subhealth posts, vector control inspector, malaria worker, municipality officer), 1 central level physician, 2 district level physicians
Focus-group discussion with hospital staff	1 group of 5	5 district level clinicians
Small discussion with community/FCHV	1 group of 2	1 community leader, 1 healthcare worker

Total number of participants	Total: 27	

**Table 2:** Dengue surveillance: activities and limitations.

Key component of recognition of dengue cases*	Extent to which activities were undertaken in Nepal in 2010	Reasons for problems identified and how to improve
Guidelines on dengue disease notification used	National clinical management guidelines available. Did not include guidelines on public health response.	Dengue not previously identified as a public health priority. New guidelines were commissioned in 2011.

Active and passive data collection	Sentinel site surveillance, only 39 sites. Not in all districts. Did not include private hospitals. Not representative of country population.	Limited funds available for widespread surveillance.

Active data collection during outbreak	Fever surveillance only occurred during the outbreak in two districts.	

Well-defined indicators for a dengue outbreak	Well-defined outbreak threshold: one or more dengue cases reported in nonendemic districts or five or more cases in endemic districts. Poor case definition of dengue.	Standard national guidelines available for dengue case definition but not consistently applied by participants.

Linking surveillance to response activities	EDCD collects and analyses the data and coordinate response measures. Poor coordination between central and periphery, confusion over which agency was in charge. No continuity of response.	Need better coordination with district offices to improve response time. Municipality meetings planned.

Training on surveillance	Despite 9 participants noted that training on surveillance was available, this was very limited and described as “not functioning at present.” Internet-based reporting had been introduced but training not provided.	More in-depth training requested by participants.

Dengue as a notifiable disease	Dengue is one of 6 notifiable diseases through the Early Warning and Response System in Nepal.	Clinicians are inexperienced with dengue and need to consider it as a differential of fever.

Appropriate level of financial resources	Budget was deemed insufficient by all participants.	

Appropriate level of human resources	More hospitals and staff need to be included in the surveillance system.	

Viral surveillance	Unable to undertake viral surveillance.	Facilities for PCR should be made available in Nepal.

Laboratory diagnostics- serological and virological	Serological tests (IgG/IgM, either RDT or ELISA) were used for diagnosis. ELISA available in “five or six centres only.” Only one participant had access to PCR (not available in general public or private facilities).	Lack of regional facilities identified as key limitation. Concern over accuracy of RDT kits.

Quality control of diagnostics.	No systematic measurement.	Introduce regional laboratory facilities to allow quality control.

Monitoring of environmental risk factors	Rainfall, temperature, and housing conditions not systematically linked into dengue surveillance system.	

*Source of key component: WHO 2009 [1].

EDCD: Epidemiology and Disease Control Division, Kathmandu, RDT: rapid diagnostic test, PCR: polymerase chain reaction, and ELISA: enzyme-linked immunosorbent assay kit.

**Table 3:** Dengue outbreak response: activities and limitations.

Key component of vector control activities*	Extent to which activities were undertaken in Nepal in 2010	Reasons for problems identified and how to improve
Entomological surveillance	Limited—few surveys were undertaken in 2006 and one in 2010 at the beginning of the outbreak, only 122 houses included in one municipality.	Due to poor human resource capacity, only two entomologists in Nepal.

Routine search and destroy of vector habitats campaign	Not in place. No routine vector control.	Dengue new and emerging, not previously seen as a threat. Coordination poor in 2010.

Emergency search and destroy of vector habitats campaign	Well run programme through community mobilisation, particularly in Chitwan and Rupandehi. Community received programme well. Not in all vulnerable areas.	Expand programmes to more vulnerable areas.

Awareness campaign	Thorough programme run by district public health office and good role of the media. Not enough IEC materials and reactive rather than anticipatory.	Need to ensure that awareness campaign is started earlier, before rainy season. Plans to include schoolchildren in future campaigns.

Water treatment by insecticide	Not done in Nepal.	Insecticides not available.

Insecticide treated nets	Not utilised in Nepal. Some nontreated nets available through malaria programme.	Funds not available.

Fogging (insecticide spraying in public areas from vehicles)	Undertaken in some districts in Nepal. Repeated several times as high public demand.	Contentious issue over effectiveness. Educate community over low effectiveness. Need advice from other organisations to maximise effectiveness.

Water container covers	Widespread use in Nepal, part of search and destroy and education campaign.	

Improvement of water supply and sanitation	Not done in Nepal. Poor coordination with district office and WASH cluster.	

Interagency coordination “Multisectoral Dengue Action Committee” setup	Poor. No NGO/INGO collaboration. Poor link and slow communication between central and periphery. Meetings held with central level and municipality, little evidence of further multisectoral action.	More municipality meetings planned.

*Source of key component: WHO 2009 [1].

IEC: information, education, communication; WASH cluster: water, sanitation, and hygiene sector; NGO/INGO: nongovernmental organisation/international nongovernmental organization.
